# Do “Virtual” and “Outpatient” Public Health Tuberculosis Clinics Perform Equally Well? A Program-Wide Evaluation in Alberta, Canada

**DOI:** 10.1371/journal.pone.0144784

**Published:** 2015-12-23

**Authors:** Richard Long, Courtney Heffernan, Zhiwei Gao, Mary Lou Egedahl, James Talbot

**Affiliations:** 1 Faculty of Medicine and Dentistry, Department of Medicine, University of Alberta, Edmonton, Alberta, Canada; 2 School of Public Health, University of Alberta, Edmonton, Alberta, Canada; 3 Clinical Epidemiology Unit, Department of Medicine, Memorial University, St. John’s, Newfoundland, Canada; 4 Alberta Health Province of Alberta, Edmonton, Alberta, Canada; McGill University, CANADA

## Abstract

**Background:**

Meeting the challenge of tuberculosis (TB) elimination will require adopting new models of delivering patient-centered care customized to diverse settings and contexts. In areas of low incidence with cases spread out across jurisdictions and large geographic areas, a “virtual” model is attractive. However, whether “virtual” clinics and telemedicine deliver the same outcomes as face-to-face encounters in general and within the sphere of public health in particular, is unknown. This evidence is generated here by analyzing outcomes between the “virtual” and “outpatient” public health TB clinics in Alberta, a province of Western Canada with a large geographic area and relatively small population.

**Methods:**

In response to the challenge of delivering equitable TB services over long distances and to hard to reach communities, Alberta established three public health clinics for the delivery of its program: two outpatient serving major metropolitan areas, and one virtual serving mainly rural areas. The virtual clinic receives paper-based or electronic referrals and generates directives which are acted upon by local providers. Clinics are staffed by dedicated public health nurses and university-based TB physicians. Performance of the two types of clinics is compared between the years 2008 and 2012 using 16 case management and treatment outcome indicators and 12 contact management indicators.

**Findings:**

In the outpatient and virtual clinics, respectively, 691 and 150 cases and their contacts were managed. Individually and together both types of clinics met most performance targets. Compared to outpatient clinics, virtual clinic performance was comparable, superior and inferior in 22, 3, and 3 indicators, respectively.

**Conclusions:**

Outpatient and virtual public health TB clinics perform equally well. In low incidence settings a combination of the two clinic types has the potential to address issues around equitable service delivery and declining expertise.

## Introduction

Tuberculosis (TB) is a preventable and curable communicable disease and therefore a public health responsibility. [1, 2] In Canada, with the exception of the First Nations and Inuit Health Branch or FNIHB (see below), there is no national TB program. Instead, each province and territory has its own public health legislation and TB prevention and care program. [3, 4] These programs face five acknowledged challenges. First, the disease is much less common than it was in the past but is now more difficult to treat because of HIV/AIDS and drug resistance. Second, the disease is concentrated in two minority groups—foreign-born persons from high incidence countries and Aboriginal peoples. [3, 5] Third, the disease while geographically focal in its spread, can occur anywhere at any time; within major metropolitan areas in selected immigrant and refugee communities and the inner city; [3] in non-major metropolitan areas in selected middle and far north Aboriginal communities, including reserve communities—parcels of land held by Canada on behalf of Indian bands. [6, 7] Four, the management of the disease is a highly collaborative enterprise involving many stakeholders. [4] And five, the management of the disease is presupposed to be equitable, a challenge given the concentration of cases in minority groups and the enormous distances over which cases may occur. [8, 9]

**Fig 1:**
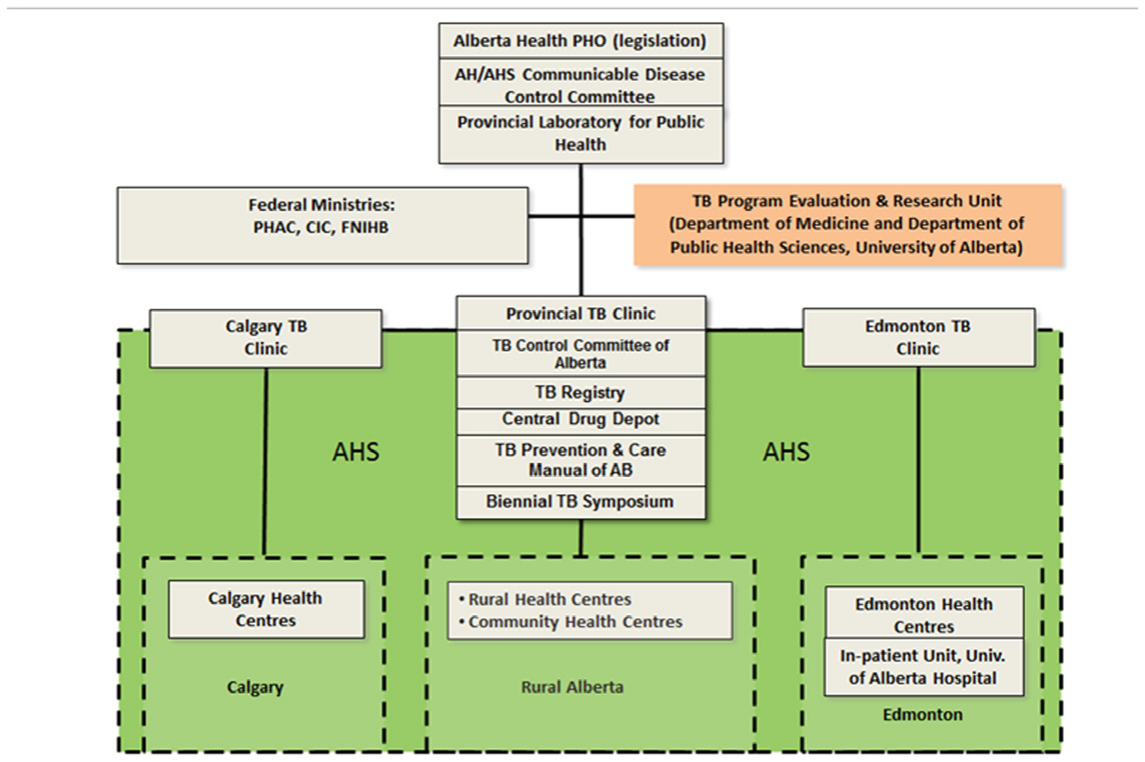
TB Prevention and Care Program of Alberta: The organization of TB services in the Province of Alberta, Canada. TB services are delivered out of three public health clinics, two outpatient (the Calgary and Edmonton TB Clinics) and one virtual. The virtual clinic also maintains the TB Registry and oversees provincial program activities. Abbreviations: PHO Provincial Health Office; AH Alberta Health; AHS Alberta Health Services; PHAC Public Health Agency of Canada; CIC Citizenship and Immigration Canada; FNIHB First Nations and Inuit Health Branch; AB Alberta.

To address these challenges, the TB program in Alberta, Canada (population 3,645,257 in 2011 [Statistics Canada]; area 661,848 km ^2^) was restructured in 1999 to be delivered out of three public health TB clinics: [4, 5] a central “virtual” clinic located in Edmonton, the capital city, and responsible for all non-major metropolitan and on-reserve First Nations cases and their contacts, and two specially ventilated “outpatient” clinics, one in each of Calgary and Edmonton, responsible for all major metropolitan cases and their contacts (see Fig 1). One outpatient clinic maintains a small satellite clinic in the inner city. The “virtual clinic” is a colloquialism in the TB program in Alberta; it refers to a clinic that does not actually see patients face-to-face. Rather, it receives from community health nurses in reserve communities or public health nurses in Rural Health Zones, referrals in the form of a medical record and chest radiograph(s) sent either electronically or by courier. Each generates a directive that is then acted upon by the referring nurse and local physicians. All active cases and their contacts are discussed at weekly rounds, which are linked as necessary to rural communities by telehealth. Public health nurses and a small group of university-based pulmonary and infectious disease physicians, whose sessional remuneration is paid by the province, staff all three clinics. Anti-TB drugs, whether for active disease or latent TB infection (LTBI), are ordered by TB physicians and dispensed from a central drug depot overseen by the virtual clinic. Medications and services are free of charge.

**Fig 2:**
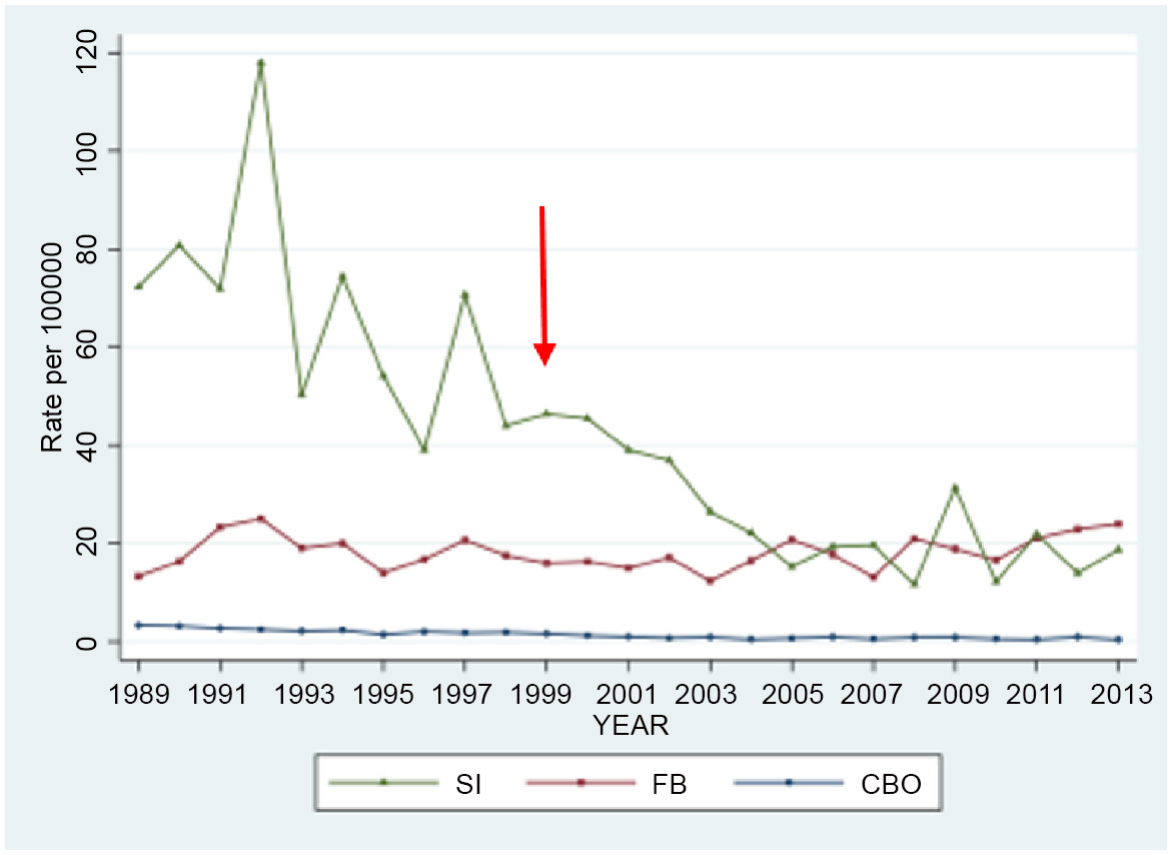
The age- and sex-adjusted incidence of tuberculosis in Alberta, 1989–2013: The age- and sex-adjusted incidence of TB in Alberta over the 25 years 1989–2013 was estimated in three population groups: Registered or Status Indians (SI), the foreign-born (FB) and Canadian-born ‘Others’ (CBO). The population estimates used in the analysis were derived from Canadian censuses conducted in 1986, 1991, 1996, 2001, and 2006. [5] After 2006 the long form of the Canadian census, which had previously estimated the foreign-born population by age and sex, was discontinued. However, it was noted that in 2001 and 2006 the proportion of the population, other than Status Indians, that was foreign-born by 5-year age and sex grouping, was relatively constant. Accordingly, the foreign-born population in 2011 was calculated on the basis of the proportions in 2006. Inter-censal estimates were estimated using linear interpolations between censuses; estimates from 2012 and 2013 were obtained by linear extrapolation. Adjustment of rates was carried out using the direct method with the total Alberta population as the reference population. The three-clinic model began in 1999; see red arrow.

Prior to 1999 one outpatient clinic served the north and the other the south of the province. Each was a product of the immediate post-sanatorium era. [10–17] The virtual clinic came into being in 1999 after a joint decision by the province and FNIHB, to conflate the separate rural components of the program. This decision was largely driven by FNIHB, a branch of Health Canada that is responsible for managing on-reserve First Nations Health, and its desire to have a more uniform approach to on-reserve TB services while minimizing patient dislocation and inconvenience. In Alberta, FNIHB contracts with the province for on-reserve TB prevention and care. Although incidence (see Fig 2) and program performance data for on-reserve First Nations before and after the establishment of the virtual clinic suggests that it may be a suitable model given the realities of TB management in Alberta and Canada, [5] there is not yet any empirical evidence of the performance of the program by clinic type. Continued government support of, or any external interest in, the three-clinic model in Alberta requires evidence of its success.

## Methods

This study was approved by the Health Research Ethics Board—Health Panel at the University of Alberta, which reviews all non-invasive health research involving patients, health information, Alberta Health Services (AHS), Edmonton Region, or Covenant Health facilities and researchers. Approval number: Pro00046503. Over a 5-year period beginning January 1^st^, 2008 all persons notified with active TB and reported to AHS, were identified in the TB Registry. Notified cases were described according to age (<15 years, 15–64 years, and >64 years), sex, population group (Canadian-born Aboriginal, Canadian-born non-Aboriginal and foreign-born), disease type (new active versus retreatment/relapse) and disease site (respiratory [with or without non-respiratory] and non-respiratory]). Canadian-born Aboriginal peoples include First Nations (Registered [Status] or non-Registered [Status] Indians according to the *Indian Act of Canada*), Métis (persons of mixed Aboriginal and non-Aboriginal ancestry who identify themselves as such on the census) and Inuit (original inhabitants of the far north who are distinct from the other Aboriginal groups in heritage language and culture). The population of non-Registered Indians and Inuit in Alberta is very small, while the population of Métis is not well defined. With very few exceptions most on-reserve Aboriginal peoples are Registered Indians.

Cases were grouped according to clinic type and performance indicators were grouped according to objective category: TB case management, TB treatment outcome and TB contact management. Virtual clinic cases that were complex could on occasion be seen in an outpatient clinic. Clinic attribution of re-located cases was based upon site of administration of the majority of active treatment. Information on performance indicators was taken from the database of the TB Registry supplemented as necessary by information from public health records and the database of the Provincial Laboratory for Public Health (PLPH). Study years and indicator selection reflects data availability and quality across clinics, existing recommendations, and the requirement that each case and contact have a minimum of one year of follow-up. [3, 9, 18–22] Indicator selection is related to outpatient care, though inpatient care may have been provided to any case for reasons of respiratory isolation, complexity of care, drug resistance/intolerance or non-adherence. Performance targets reflect those recommended by best practice or official bodies. [3, 9, 18, 23] The three groups of indicators in the two clinic types are compared to measure performance. The same indicators are also compared in virtual clinic cases according to place-of-residence, on-reserve versus off-reserve.

i) TB case management indicators: Ten indicators are assessed. One indicator, the proportion of all TB cases who were culture-positive, is a surrogate for the thoroughness of clinical investigation and diagnostic specimen handling. Two related indicators tracked the processing of positive cultures: the proportion of all *Mycobacterium tuberculosis* isolates that underwent drug susceptibility testing (DST) and genotyping in the PLPH. Another indicator, the proportion of all TB cases who were aged <5 years, is a surrogate measure of the extent of household transmission to a vulnerable population. [24] The other six case management indicators, HIV testing of TB patients and the response of airway secretion smear-positive pulmonary TB cases to treatment, are recommended by the Canadian TB Standards and official statements of the Public Health Agency of Canada. [3, 9, 25] Sputum samples were collected, and chest radiographs performed within two weeks of completion of the initial and one month of completion of the continuation phase, respectively.

ii) TB treatment outcome indicators: Six indicators are assessed: the proportion of all TB cases treated with directly-observed therapy (DOT); the proportion of all culture-positive TB cases that relapsed within two years of completing treatment; the proportion of all culture-positive TB cases who died with TB the primary or contributory cause of death; [26, 27] the proportion of all Canadian-born culture-positive TB cases with first-line drug resistance—an indication of the quality of treatment and treatment adherence; and the proportion of smear-positive PTB cases with anti-TB drugs started within 72 hours (equivalent to one weekend plus one statutory holiday) of a positive PCR (polymerase chain reaction or nucleic acid amplification test) and who completed treatment within 12 months of treatment start.

iii) TB contact management indicators: Twelve indicators (six indicators in two age groups) are assessed in close contacts of two different random samples of smear-positive PTB cases managed in the two types of clinics. One indicator assessed the proportion of close contacts aged <5 and ≥5 years, of smear-positive PTB cases who were completely assessed, that is had a symptom inquiry and tuberculin skin test (TST) 8–12 weeks post-final contact with the smear-positive case if not already determined to be TST positive, a chest radiograph if determined to be symptomatic or to have a positive TST, and sputum for acid-fast bacilli smear and culture if determined to be symptomatic or have an abnormal chest radiograph. Two indicators sought to expose diagnostic delay: the number of assessed contacts, again stratified by age, who were secondary cases as described elsewhere, [28] and the number of new positive TSTs defined as those with a positive TST and no past positive TST and those with TST conversion, as defined by the Canadian TB Standards, [3] per completely assessed close contacts of smear-positive cases. Three indicators report on the recommendation, acceptance and completion of treatment of LTBI among close contacts of smear-positive cases with a new positive TST. If the true positive nature of a TST was in doubt and an interferon-gamma release assay was performed, the results of the latter were considered definitive.

### Statistical Analysis

Counts/proportions are provided and significant differences between groups tested by the t-test and chi-square test, respectively. If the expected count was lower than 5, exact p-value was calculated; p< 0.05 was considered significant. Stratified random sampling by population group was used to pick the smear-positive PTB cases in the virtual and outpatient clinics whose close contacts were evaluated.

## Results

There were 841 patients diagnosed with TB in the Province of Alberta in 2008–2012 of which 150 (18%) were managed in the virtual clinic and 691 (82%) were managed in the outpatient clinics (see Table 1). Cases managed in the virtual and outpatient clinics did not differ by age, sex, or disease type. Compared to outpatient clinic cases virtual clinic cases were significantly (p = 0.0001) more likely to be Aboriginal peoples (47% vs 4%) and less likely to be foreign-born (41% vs 87%). Compared to virtual clinic cases outpatient clinic cases were significantly (p = 0.02) more likely to have non-respiratory disease alone (34% vs 24%), a finding consistent with the higher proportion of foreign-born cases in the outpatient clinics and the known proclivity for foreign-born cases to have non-respiratory TB. [3] Of the total of 237 adult (age >14 years) smear-positive PTB cases reported in the province between 2008 and 2012, 50 (21%) were managed in the virtual clinic and 187 (79%) were managed in the outpatient clinics.

**Table 1:** Tuberculosis Case Characteristics by Clinic Site of Care, Alberta, 2008–2012.

	Clinic Site of Care*			
Characteristic	All Clinics	Virtual Clinic	Outpatient Clinics	p-value
	n (%)	n (%)	n (%)	
**No. Assessed**	841	150 (18)	691 (82)	NA
**Age (Years)**				0.78
<15	49 (6)	10 (7)	39 (6)	
15–64	613 (73)	106 (71)	507 (73)	
>64	179 (21)	34 (23)	145 (21)	
**Sex**				0.56
Male	464 (55)	86 (57)	378 (55)	
Female	377 (45)	64 (43)	313 (45)	
**Population Group**				0.0001
CBA	100 (12)	70 (47)	30 (4)	
CBO	81 (10)	19 (13)	62 (9)	
FB	660 (78)	61 (41)	599 (87)	
**Disease Type**				0.45
New Active	791 (94)	139 (93)	652 (94)	
Retreatment/Relapse	50 (6)	11 (7)	39 (6)	
**Disease Site** ^†^				
**Respiratory**	571 (68)	114 (76)	457 (66)	0.65
S+ C+	237 (41)	50 (44)	187 (41)	
S‒ C+	244 (43)	49 (43)	195 (43)	
C‒	90 (16)	15 (13)	75 (16)	
**Non-Respiratory**	270 (32)	36 (24)	234 (34)	0.02
C+	228 (84)	32 (89)	196 (84)	
C‒	42 (16)	4 (11)	38 (16)	

Abbreviations: CBA Canadian-born Aboriginal; CBO Canadian-born Other; FB Foreign-born; S+ smear-positive; S‒ Smear-negative; C+ culture-positive; C‒ culture-negative

* See text for definition of clinic type

^†^ All smear-positive cases had semi-quantitative smears of 1+ or greater

Within the ten case management indicators, both clinic types achieved similarly good or acceptable target proportions of cases HIV tested, cases <5 years of age, cases culture-positive for *M*. *tuberculosis*, and cases with isolates that underwent DST and genotyping (see Table 2). In the virtual and outpatient clinics respectively, 97% and 94% of adult (age >14 years) respiratory cases were culture-positive (p = 0.21) and 96% and 96% of adult TB cases aged 15–64 years, the age group that includes the vast majority of co-infected patients, were HIV tested (p = 0.80), data not shown. In both types of clinics similarly sub-optimal target proportions of smear-positive PTB cases had their mycobacteriologic and radiographic response to treatment, monitored. With respect to being followed to three smears negative the virtual clinic performed better than the outpatient clinics (93% vs 55%, p = 0.0001). With respect to having an end-of-initial phase sputum culture and chest radiograph, the outpatient clinics performed better than the virtual clinic (78% vs 50%, p = 0.01; 68% vs 52%, p = 0.05, respectively). Similar proportions of virtual and outpatient clinic cases had an end-of-continuation phase sputum culture and chest radiograph.

**Table 2:** Tuberculosis Case Management Indicators by Clinic Site of Care, Alberta, 2008–2012.

		Clinic Site of Care*			
Indicator	Target	All Clinics	Virtual Clinic	Outpatient Clinics	p-value
	(%)	n (%)	n (%)	n (%)	
**TB Cases**		**841**	**150**	**691**	
HIV tested^†^	>90	798 (95)	145 (99)	653 (95)	0.33
			[3]	[1]	
Pediatric cases <5 years of age	<5	21 (3)	5 (3)	16 (2)	0.56
**Culture-positive TB cases**	75–85	709 (84)	131 (87)	578 (84)	0.26
DST results reported	100	701 (99)	131 (100)	570 (99)	0.36
Genotype reported	100	694 (98)	130 (99)	564 (98)	0.33
**Smear-positive PTB cases** ^‡^		**237**	**50**	**187**	
Followed to 3 smears negative	100	142 (63)	43 (93)	99 (55)	0.001
			[4]	[7]	
End of initial phase culture	90	163 (72)	23 (50)	140 (78)	0.01
			[4]	[8]	
End of initial phase CXR	100	147 (65)	24 (52)	123 (68)	<0.05
			[4]	[6]	
End of continuation phase culture	50	98 (46)	25 (58)	73 (43)	0.07
			[7]	[17]	
End of continuation phase CXR	100	175 (83)	32 (76)	143 (84)	0.23
			[8]	[17]	

Abbreviations: DST drug susceptibility test; PTB pulmonary tuberculosis; HIV human immunodeficiency virus; CXR chest x-ray

* See text for definition of clinic type and phase of treatment

^†^ Patients who were diagnosed at death may not have been HIV tested. If not they are listed in square brackets beneath the indicator result and excluded from the denominator.

^‡^ Patients who died or were transferred out in the interim are listed in square brackets beneath the indicator result and excluded from the denominator

Within the six treatment outcome indicators (see Table 3) the virtual and outpatient clinics performed similarly well with low proportions of culture-positive cases relapsing within two years or dying a TB-related death—though the virtual clinic was off-target, and similarly low proportions of Canadian-born culture-positive cases with initial drug resistance. Similarly high proportions of smear-positive PTB cases started treatment within 72 hours of PCR and completed treatment within 12 months of starting. With respect to the proportion of TB cases treated with DOT the virtual clinic performed better than the outpatient clinics (100.0% vs 95.0%, p = 0.004).

**Table 3:** Tuberculosis Treatment Outcome Indicators by Clinic Site of Care, Alberta, 2008–2012.

		Clinic Site of Care*			
Indicator	Target	All Clinics	Virtual Clinic	Outpatient Clinics	p-value
	(%)	n (%)	n (%)	n (%)	
**TB cases**		**841**	**150**	**691**	
Number Tx with DOT†	100	789 (96)	145 (100)	644 (95)	0.004
		[15]	[5]	[10]	
**Culture-positive TB cases**		**709**	**131**	**578**	
Number relapsed within 2 years^‡^	<3	3 (<1)	0 (0)	3 (1)	1.00
		[68]	[13]	[55]	
Number with TB-related death^§^	<5	24 (4)	8 (6)	16 (3)	0.063
		[4]	[5]	[19]	
**CB culture-positive TB cases**		**138**	**80**	**58**	
Number with initial drug resistance	<4	3 (2)	2 (3)	1 (2)	1.00
		[0]	[0]	[0]	
**Smear-positive PTB cases**		**237**	**50**	**187**	
Number Tx within 72 hr. of PCR^†^	>90	206 (88)	41 (85)	165 (89)	0.32
		[4]	[2]	[2]	
Number completing Tx within 12 mo.^‡^	≥95	199 (95)	39 (93)	160 (96)	0.42
		[28]	[8]	[20]	

Abbreviations: TB tuberculosis; Tx treatment; DOT Directly-Observed Therapy; CB Canadian-born; PTB pulmonary tuberculosis; PCR Polymerase Chain Reaction

* See text for definition of clinic type

^†^ Those who died without treatment are listed in square brackets beneath the indicator result and excluded from the denominator

^‡^ Those who were TB deaths or transferred out are listed in square brackets beneath the indicator result and excluded from the denominator

^§^ Those who died without treatment or transferred out are listed in square brackets beneath the indicator result and excluded from the denominator

Summarized in Tables 4 and 5 is the performance of the two types of clinics with respect to the management of close contacts of randomly selected smear-positive PTB cases. Random selection was performed twice to minimize possible bias caused by random sampling. Contacts were stratified by age into those <5 and ≥5 years of age. Virtual clinic cases had on average more contacts per case than outpatient clinic cases, which may indicate overcrowding in some reserve communities. [29] On balance, both clinic types achieved similarly good or acceptable target proportions for most indicators. In one random sampling (4) the outpatient clinics performed better than the virtual clinic when it came to the number of close contacts ≥5 years of age accepting treatment of LTBI after being recommended (92.3% vs 70.0%, p = 0.016). In the other random sampling (5) the virtual clinic performed better than the outpatient clinics when it came to the number of close contacts that were completely assessed (78.4% vs 63.1, p = 0.02).

**Table 4:** Tuberculosis Contact Management indicators by Clinic Site of Care, Alberta, 2008–2012 (Random draw 1).

		Clinic Site of Care*			
Indicator	Target	All Clinics	Virtual Clinic	Outpatient Clinics	p-value^§^
	(%)	n	n	n	
**Randomly selected PTB cases**		71	36	35	
**Close contacts**					
< 5 yr (%)		95	74 (9.7)	21(8.6)	0.74
≥ 5 yr (%)		771	592 (90.3)	179 (91.4)	0.74
**A,No. close contacts assessed** ^†^					
< 5 yr (%)	100	90 (95)	71 (96.4)	19 (88.6)	0.31
≥ 5 yr (%)	80	533 (71)	426 (79.4)	107 (69.8)	0.17
**B,No. secondary cases/A**					
< 5 yr (%)	1–2	1 (3)	0 (0.0)	1 (3.3)	0.16
≥ 5 yr (%)	1–2	14 (2)	11 (2.2)	3 (4.5)	0.49
**C,No. new positive TST's** ^‡^ **/A**					
< 5 yr (%)	31–36	14 (100)	14 (20.1)	0 (0.0)	0.06
≥ 5 yr (%)	31–36	167 (16)	122 (35.0)	45 (42.5)	0.46
**D,No. recommended Tx LTBI/C**					
< 5 yr (%)	100	14 (100)	14 (100)	0 (NA)	NA
≥ 5 yr (%)	100	159 (93)	117 (93.8)	42 (87.8)	0.44
**E,No. accepting Tx LTBI/D**					
< 5 yr (%)	100	14 (100)	14 (100)	0 (NA)	
≥ 5 yr (%)	80	127 (80)	89 (70.0)	38 (92.3)	0.016
**F,No. completing Tx LTBI/E**					
< 5 yr (%)	100	14 (100)	14 (100)	0 (NA)	
≥ 5 yr (%)	80	95 (76)	69 (76.3)	26 (62.6)	0.27

Abbreviations: PTB Pulmonary TB; yr year; TST tuberculin skin test; Tx treatment, LTBI latent TB infection

* See text for definition of clinic type

^†^ The mean proportions (%) reported for each indicator take into account the number of contacts per smear-positive case.

^**‡**^ New positive TST's include those with a new positive TST and no past positive TST and those with TST conversion. They do not include secondary cases

^**§**^Adjusted for population group.

**Table 5:** Tuberculosis Contact Management indicators by Clinic Site of Care, Alberta, 2008–2012. (Random draw 2).

		Clinic Site of Care*			
Indicator	Target	All Clinics	Virtual Clinic	Outpatient Clinics	p-value^§^
	(%)	n	n	n	
**Randomly selected PTB cases**		71	36	35	
**Close contacts**					
< 5 yr (%)		74	49 (6.7)	25 (5.9)	0.77
≥ 5 yr (%)		957	620 (93.3)	337 (94.1)	0.77
**A,No. close contacts assessed** ^†^					
< 5 yr (%)	100	71 (95)	48 (99.7)	23 (84.4)	0.09
≥ 5 yr (%)	80	673 (71)	455 (78.4)	218 (63.1)	0.02
**B,No. secondary cases/A**					
< 5 yr (%)	1–2	3 (3)	1 (7.1)	2 (6.5)	0.95
≥ 5 yr (%)	1–2	12 (2)	9 (2.1)	3 (4.5)	0.47
**C,No. new positive TST's** ^‡^ **/A**					
< 5 yr (%)	31–36	10 (100)	10 (14.4)	0 (0.0)	0.18
≥ 5 yr (%)	31–36	184 (16)	125 (37.0)	59 (40.1)	0.77
**D,No. recommended Tx LTBI/C**					
< 5 yr (%)	100	10 (100)	10 (100)	0 (NA)	NA
≥ 5 yr (%)	100	170 (93)	120 (94.2)	50 (80.4)	0.08
**E,No. accepting Tx LTBI/D**					
< 5 yr (%)	100	10 (100)	10 (100)	0 (NA)	NA
≥ 5 yr (%)	80	132 (80)	88 (67.7)	44 (85.9)	0.06
**F,No. completing Tx LTBI/E**					
< 5 yr (%)	100	10 (100)	10 (100)	0 (NA)	NA
≥ 5 yr (%)	80	105 (76)	72 (79.5)	33 (69.0)	0.36

Abbreviations: PTB pulmonary TB; yr year; TST tuberculin skin test; Tx treatment, LTBI latent TB infection

* See text for definition of clinic type

^†^ The mean proportions (%) reported for each indicator take into account the number of contacts per smear-positive case.

^**‡**^ New positive TST's include those with a new positive TST and no past positive TST and those with TST conversion. They do not include secondary cases

^**§**^Adjusted for population group.

Among the 150 cases managed by the virtual clinic, 39 (26%) were living on-reserve and 111 (74%) were living off-reserve. Of those living on-reserve 39 (100%) were Registered Indians. Of those living off-reserve, 61 (55%) were foreign-born, 31 (28%) were Canadian-born Aboriginal (19 [61%] Registered Indian, 3 [10%] non-Registered Indian and 9 [29%] Métis) and 19 (17%) were Canadian-born non-Aboriginal. Of those living on-reserve and off-reserve 18 (46%) and 32 (29%), respectively, had smear-positive PTB. In Tables 6, 7, 8 and 9 the above 28 performance indicators are compared in virtual clinic cases that were living on-reserve versus virtual clinic cases that were living off-reserve. There were no significant differences by place of residence on any indicator.

**Table 6:** Virtual Clinic Tuberculosis Case Management Indicators by Place of Residence, On-Reserve versus Off-Reserve of Case-Patients, Alberta, 2008–2012.

		On-Reserve versus Off-Reserve*			
Indicator	Target	On & Off	On	Off	p-value
	(%)	n (%)	n (%)	n (%)	
**TB Cases**		**150**	**39**	**111**	
HIV tested^†^	>90	145	36 (97)	109 (98)	0.44
			[2]	[0]	
Pediatric cases <5 years of age	<5	5	1 (3)	4 (4)	1.00
**Culture-positive TB cases**	75–85	131	35 (90)	96 (86)	0.78
DST results reported	100	131	35 (100)	96 (100)	1.00
Genotype reported	100	130	35 (100)	95 (99)	1.00
**Smear-positive PTB cases** ^‡^		**50**	**18**	**32**	
Followed to 3 smears negative	100	43	16 (100)	27 (90)	0.54
			[2]	[2]	
End of initial phase culture	90	23	7 (43)	16 (53)	0.54
			[2]	[2]	
End of initial phase CXR	100	24	7 (44)	17 (57)	0.40
			[2]	[2]	
End of continuation phase culture	50	25	9 (60)	16 (57)	0.86
			[3]	[4]	
End of continuation phase CXR	100	30	10 (67)	20 (71)	0.74
			[3]	[4]	

Abbreviations: DST drug susceptibility test; PTB pulmonary tuberculosis; HIV human immunodeficiency virus; CXR Chest x-ray

* See text for definition of phase of treatment

^†^ Patients who were diagnosed at death may not have been HIV tested. If not they are listed in square brackets beneath the indicator result and excluded from the denominator

^‡^ Patients who died or were transferred out in the interim are listed in square brackets beneath the indicator result and excluded from the denominator

**Table 7:** Virtual Clinic Tuberculosis Treatment Outcome Indicators by Place of Residence, On-Reserve versus Off-Reserve of Case-Patients, Alberta, 2008–2012.

		On-Reserve versus Off-Reserve			
Indicator	Target	On & Off	On	Off	p-value
	(%)	n (%)	n (%)	n (%)	
**TB Cases**		**150**	**39**	**111**	
Number Tx with DOT*	100	145 (100)	36 (100)	109 (100)	0.33
		[5]	[3]	[2]	
**Culture-positive TB cases**		**131**	**35**	**96**	
Number relapsed within 2 years†	<3	0 (0)	0 (0)	0 (0)	1.00
		[13]	[7]	[6]	
Number with TB-related death*	<5	8 (6)	3 (9)	5 (5)	0.42
		[5]	[3]	[2]	
**CB culture-positive TB cases**		**80**	**35**	**45**	
Number with initial drug resistance	<4	2 (3)	0 (0)	2 (4)	0.50
		[0]	[0]	[0]	
**Smear-positive PTB cases**		**50**	**18**	**32**	
Number Tx within 72 hr. of PCR*	>90	41 (85)	13 (81)	28 (88)	0.67
		[2]	[2]	[0]	
Number completing Tx within 12 mo.^†^	≥95	39 (92)	13 (93)	26 (93)	1.00
		[8]	[4]	[4]	

Abbreviations: TB Tuberculosis; Tx Treatment; DOT Directly-Observed Therapy; NA not applicable; CB Canadian-born; PTB pulmonary TB; PCR Polymerase Chain Reaction

*Those who died without treatment are listed in square brackets beneath the indicator result and excluded from the denominator

^†^ Those who were TB deaths or transferred out are listed in square brackets beneath the indicator result and excluded from the denominator

**Table 8:** Virtual Clinic Tuberculosis Contact Management indicators by place of residence—on versus off reserve—Alberta, 2008–2012 (Random draw 1).

		Place of Residence			
Indicator	Target	All Communities	Virtual On	Virtual Off	p-value
	(%)	n	n	n	
**Randomly selected PTB cases**		**36**	**10**	**26**	
**Close contacts**					
< 5 yr (%)		74	53 (12.8)	21(8.6)	0.39
≥ 5 yr (%)		592	198 (87.2)	394 (91.4)	0.39
**No. close contacts assessed** *					
< 5 yr (%)	100	71 (96)	51 (97.1)	20 (96.2)	0.88
≥ 5 yr (%)	80	426 (76)	148 (75.0)	278 (81.1)	0.47
**No. secondary cases**					
< 5 yr (%)	1–2	0 (0)	0 (0.0)	0 (0.0)	NA
≥ 5 yr (%)	1–2	11 (2)	8 (4.3)	3 (1.4)	0.17
**No. new positive TST's** ^†^					
< 5 yr (%)	31–36	14 (21)	9 (15.7)	5 (21.8)	0.72
≥ 5 yr (%)	31–36	122 (16)	32 (25.9)	90 (38.5)	0.35
**No. recommended Tx LTBI**					
< 5 yr (%)	100	14 (100)	9 (100)	5 (100)	NA
≥ 5 yr (%)	100	117 (93)	31 (83.3)	86 (96.9)	0.15
**No. accepting Tx LTBI**					
< 5 yr (%)	100	14 (100)	9 (100)	5 (100)	NA
≥ 5 yr (%)	80	89 (75)	27 (91.5)	62 (64.6)	0.10
**No. completing Tx LTBI**					
< 5 yr (%)	100	14 (100)	9 (100)	5 (100)	NA
≥ 5 yr (%)	80	69 (80)	22 (90.0)	47 (72.3)	0.33

Abbreviations: Pop-Grp population group; PTB pulmonary TB; yr year; TST tuberculin skin test; Tx treatment, LTBI latent TB infection

*The mean proportions (%) reported for each indicator take into account the number of contacts per smear-positive case.

^**†**^New positive TST's include those with a new positive TST and no past positive TST and those with TST conversion. They do not include secondary cases

**Table 9:** Virtual Clinic Tuberculosis Contact Management indicators by place of residence—on versus off reserve—Alberta, 2008–2012 (Random draw 2).

		Place of Residence			
Indicator	Target	All Communities	Virtual On	Virtual Off	p-value
	(%)	n	n	n	
**Randomly selected PTB cases**		**36**	**11**	**25**	
**Close contacts**					
< 5 yr (%)		49	34 (5.3)	15 (7.3)	0.64
≥ 5 yr (%)		620	243 (94.7)	377 (92.7)	0.64
**No. close contacts assessed** *					
< 5 yr (%)	100	48 (96)	33 (99.1)	15 (100.0)	0.08
≥ 5 yr (%)	80	455 (76)	199 (80.1)	256 (77.6)	0.76
**No. secondary cases**					
< 5 yr (%)	1–2	1 (1)	1 (25.0)	0 (0.0)	0.08
≥ 5 yr (%)	1–2	9 (2)	7 (3.8)	2 (1.4)	0.22
**No. new positive TST's** ^†^					
< 5 yr (%)	31–36	10 (21)	7 (4.6)	3 (18.3)	0.40
≥ 5 yr (%)	31–36	125 (16)	35 (25.5)	90 (42.0)	0.19
**No. recommended Tx LTBI**					
< 5 yr (%)	100	10 (100)	7 (100)	3 (100)	NA
≥ 5 yr (%)	100	120 (93)	34 (87.5)	86 (96.9)	0.25
**No. accepting Tx LTBI**					
< 5 yr (%)	100	10 (100)	7 (100)	3 (100)	NA
≥ 5 yr (%)	80	88 (75)	27 (76.7)	61 (64.6)	0.40
**No. completing Tx LTBI**					
< 5 yr (%)	100	10 (100)	7 (100)	3 (100)	NA
≥ 5 yr (%)	80	72 (80)	26 (97.1)	46 (72.3)	0.10

Abbreviations: Pop-Grp population group; PTB pulmonary TB; yr year; TST tuberculin skin test; Tx treatment, LTBI latent TB infection

* The mean proportions (%) reported for each indicator take into account the number of contacts per smear-positive case.

^**†**^ New positive TST's include those with a new positive TST and no past positive TST and those with TST conversion. They do not include secondary cases

## Discussion

The performance of a TB prevention and care program delivered in its entirety out of three public health clinics, one virtual and two outpatient, was assessed. The assessment, overall and by clinic type, included reference to 16 case management and treatment outcome indicators and 12 contact management indicators. It did not include an assessment of the screening of immigration referrals, which is already known to be good in Alberta (province-wide in 2010, 92.6% of referrals were completely assessed [unpublished data]), or persons with high-risk medical conditions. Overall performance was good and although not functioning completely independent of one another, there was no evidence that the performance of one clinic type was systematically superior or inferior to the other. On 22 indicators performance was not significantly different between the clinics; on 3 indicators, one related to case management, one related to treatment outcome and one related to contact management, virtual clinic performance was superior; on 3 indicators, two related to case management and one related to contact management, virtual clinic performance was inferior. With respect to the mycobacteriologic and radiographic response to treatment both clinics performed below target for most indicators suggesting the need for program-wide improvements. With respect to TB deaths the virtual clinic exceeded the target rate, no doubt a reflection of its higher Aboriginal case load and the known high TB case-fatality rate in Aboriginal peoples in Alberta, the cause of which is almost certainly multi-factorial. [27] Further discussion of the rationale behind the three-clinic model and its performance by population group follows.

The organization of Alberta’s TB prevention and care program out of three public health clinics, and as an academic physician/public health nurse collaboration, was in response to the operational challenges summarized in the introduction and FNIHB’s expressed concerns about uniformity of approach, and reducing patient dislocation and inconvenience. Expert physician services, coupled to public health nursing, recognize that consideration of the medical and social context is essential, [2] and create an environment conducive to program evaluation and research. In both clinic types the pairing of public health expertise with an unwritten line of authority—primary care physicians are copied on related communications and otherwise permit the program to work through them—operationalizes the fundamental understanding that case and contact management is both a medical and public health action. [30–33] Effective treatment of the smear-positive case, the first objective of TB care, not only relieves symptoms and provides a lasting cure, but interrupts transmission and prevents resistance. The establishment of the virtual clinic in 1999 united the separate rural components of the program by providing centralized expertise to sparsely populated areas, especially important as case load falls, and enabling ‘management-in-place’ of on-reserve First Nations. In addition to recognizing the negative history attached to the removal of First Nations to distant sanatoria, it resembles the neighborhood clinics described by Curry, serving patients who are often at a lower socio-economic level, less well educated and living in overcrowded substandard housing. [11, 14] Virtual clinic performance on- and off-reserve was no different and, while two major outbreaks were reported in on-reserve First Nations between 1989 and 1998 none have been reported since. [34]

The TB prevention and care program in Alberta may be said to be categorical in the sense that it is unambiguously explicit or direct. [35, 36] Each clinic is a dedicated public health clinic. Each is solely funded by the Provincial Government, with the exception of the virtual clinic which is co-funded by FNIHB. Outside of the support from FNIHB there is no direct federal funding of provincial/territorial TB programs in Canada such as exists in the United States, [35–37] though indirect support is provided through the services of: (i) the Public Health Agency of Canada; (ii) Citizenship and Immigration Canada; (iii) the National Reference Laboratory and; (iv) Correctional Services Canada. [3] By the ultimate outcome measure—fewer cases of the disease or a decline or accelerated decline in incidence, [38] the centralized three-clinic model in Alberta has achieved good results in the Canadian-born population but disappointing results in the foreign-born population (see Fig 2). Canada-wide since 1999, TB rates have fallen in Canadian-born non-Aboriginals and to a lesser extent the foreign-born, but have remained unchanged or increased in Aboriginal groups. [39, 40] The failure of rates to have fallen in the foreign-born in Alberta is complicated, and will require a program-wide strategic change.

Normally, good case and contact management will result in a reduced reservoir of LTBI, subsequent numbers of cases and incidence of TB. [41] In Alberta, any success in this regard is plainly offset by the extent to which immigration, largely driven be employment opportunities, replenishes the reservoir. Between 1989 and 2003 the province accepted an average of 15,497 new immigrants per year; between 2004 and 2012, an average of 25,376 per year. [42] Over this last 9 year period 73.1% of new immigrants arrived from either the Africa or Middle East source area or the Asia and Pacific source area. [43] Depending upon the incidence of TB in their country of birth immigrants are more or less likely to have LTBI and to reactivate after arrival. [44, 45] Once the proportion of total cases that are foreign-born in high income countries exceeds 70%, it is known that very little change in incidence (2% or less per year) can be expected without either; (i) improved TB prevention and care programs overseas, or (ii) expanded in-country prevention and care activities. [46] In Alberta, between 2008 and 2012 the foreign-born accounted for an average of 78.5% (range 70.4%to 83.5%) of the total cases. That the incidence of TB in the Canadian-born in Alberta continues to fall in spite of rising numbers of foreign-born cases, is further evidence against significant transmission between these groups. [47, 48]

In summary, in response to epidemiologic, geographic, and case management realities, together with the imperative to provide equitable care, a wholly public health—academic TB service delivery model has evolved in Alberta, Canada. Measured in program performance this model has been very successful. In the pre-elimination/elimination phase of TB it has the potential to address issues of declining expertise and quality of care.
